# A Novel Online Calculator Predicting Acute Kidney Injury After Liver Transplantation: A Retrospective Study

**DOI:** 10.3389/ti.2023.10887

**Published:** 2023-01-19

**Authors:** Jianfeng Zeng, Qiaoyun Li, Qixing Wu, Li Li, Xijiu Ye, Jing Liu, Bingbing Cao

**Affiliations:** ^1^ Department of Anesthesiology, The Sun Yat-sen Memorial Hospital of Sun Yat-sen University, Guangzhou, China; ^2^ Department of Physiology, The Zhongshan Medical School of Sun Yat-sen University, Guangzhou, China; ^3^ Department of Anesthesiology, The First Affiliated Hospital University of Science and Technology of China, Hefei, China; ^4^ Department of Anesthesiology, The Eighth Affiliated Hospital of Sun Yat-sen University, Shenzhen, China

**Keywords:** liver transplantation, risk factors, acute kidney injury, nomogram, online calculator

## Abstract

Acute kidney injury (AKI) after liver transplantation (LT) is a common complication, and its development is thought to be multifactorial. We aimed to investigate potential risk factors and build a model to identify high-risk patients. A total of 199 LT patients were enrolled and each patient data was collected from the electronic medical records. Our primary outcome was postoperative AKI as diagnosed and classified by the KDIGO criteria. A least absolute shrinkage and selection operating algorithm and multivariate logistic regression were utilized to select factors and construct the model. Discrimination and calibration were used to estimate the model performance. Decision curve analysis (DCA) was applied to assess the clinical application value. Five variables were identified as independent predictors for post-LT AKI, including whole blood serum lymphocyte count, RBC count, serum sodium, insulin dosage and anhepatic phase urine volume. The nomogram model showed excellent discrimination with an AUC of 0.817 (95% CI: 0.758–0.876) in the training set. The DCA showed that at a threshold probability between 1% and 70%, using this model clinically may add more benefit. In conclusion, we developed an easy-to-use tool to calculate the risk of post-LT AKI. This model may help clinicians identify high-risk patients.

## Graphical Abstract

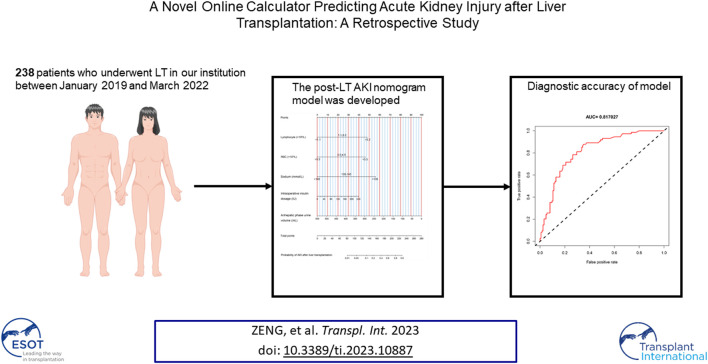

**Clinical Trial Registration:** chictr.org.cn, identifier ChiCTR2200059927.

## Introduction

Liver transplantation (LT) is the only definitive treatment for patients with end-stage liver disease, and the number of patients receiving LT has increased significantly during recent decades (1). Acute kidney injury (AKI) is a frequent but severe complication after LT and is associated with increased morbidity (2, 3). According to the current literature, which applied the 2012 Kidney Disease Improving Global Outcomes (KDIGO) criteria, the incidence of post-LT AKI is high, ranging from approximately 40%–60% (2, 4–8). For LT patients, there is no doubt that postoperative AKI is strongly associated with poor clinical outcomes, including increased graft rejection, longer hospital stay, more healthcare costs and shorter overall survival (2, 3). To avoid or reduce the occurrence of AKI after LT, discovering predisposing factors that can help us to identify high-risk patients is a pressing need.

The development of post-LT AKI is thought to be multifactorial (3). Several preoperative, intraoperative and postoperative variables have been reported, including serum creatinine, graft characteristics, intraoperative hemorrhage, urine output, cold ischemic time, calcineurin inhibitor nephrotoxicity and postoperative intensive care unit (ICU) stay (6, 9–11). Unfortunately, many studies used different postoperative AKI criteria and lack a standard definition; therefore, the above risk variables may not be sufficiently reliable among different studies. On the other hand, as we continue to learn more about the pathogenesis of post-LT AKI, new technique application and individualized treatment improved, and more new factors were identified. For instance, Pulitano and colleagues (12) confirmed that serum endothelin-1 and interleukin-18 levels were significantly predictive of post-LT AKI, which are involved in liver ischemia reperfusion injury (IRI).

A previous study reported that early diagnosis and treatment may improve the outcome of post-LT AKI patients (2). Therefore, constructing an accurate model to predict post-LT AKI becomes particularly meaningful. In this study, we applied the KDIGO criteria to define AKI after LT, identified potential risk factors and then developed a predictive model. We further constructed an easy-to-use online calculator to facilitate the calculation of the probability of AKI at the end of the transplant procedure. The objectives of our study are to help clinicians identify high-risk AKI patients, facilitate clinical decision-making and improve the prognosis of LT patients.

## Patients and Methods

### Study Population

Inclusion criteria were patients who underwent LT in our institution between January 2019 and March 2022. Exclusion criteria were as following: 1) patients who were less than 18 years old; 2) received continuous renal replacement therapy (CRRT); 3) diagnosed with kidney diseases before surgery (including hepato-renal syndrome); 4) died within 48 h after surgery; 5) underwent combined liver-kidney transplantation or retransplantation and 6) missing important data. After the surgery, all patients were followed up daily until the discharge time. Besides, urine output and serum creatinine were used for routinely evaluation of renal function. This manuscript was prepared for publication using the applicable Equator guidelines for quality improvement studies.

### Data Collection

We manually extracted preoperative, intraoperative and postoperative data from the electronic medical records by two independent investigators. Then, the patient cohort was randomly divided into two parts at a ratio of 7:3 for training and validation. Of note, we only used preoperative and intraoperative data to construct the model, and postoperative data were only applied to define the end point and outcome analysis. Besides, we failed to extract warm and cold ischemic time as the two variables were not available in our electronic systems. In this study, the liver was almost retrieved from deceased after brain death (DBD) donors.

The preoperative data included demographic information, preoperative comorbidities, etiologies, clinical manifestations, laboratory data, and hemodynamic parameters. Demographic information included sex, age, body mass index (BMI), American Society of Anesthesiologists (ASA) score and personal lifestyle habits (smoking and alcoholism). The preoperative comorbidities included diabetes mellitus, hypertension, cardiovascular diseases (e.g., coronary heart disease) and respiratory diseases (e.g., chronic obstructive pulmonary disease). Etiologies of the studied population included viral hepatitis (e.g., hepatitis B virus), liver cancer (e.g., hepatocellular carcinoma) and others (e.g., alcohol-related liver disease and cholestatic liver disease). The clinical manifestations included symptoms of fluid overload (e.g., ascites) and major organ dysfunction (e.g., encephalopathy). The extent of ascites was graded according to the maximum depth of pelvic ascites measured by ultrasound: no ascites, non-severe ascites (<10 cm), and severe ascites (≥10 cm). Preoperative laboratory data included blood biochemistry, routine blood tests, and coagulation function, which was defined as the last measurement before surgery. The hemodynamic parameters included central venous pressure (CVP) and cardiac output (CO) on the day before surgery. We measured CVP *via* a central venous catheter, while the cardiac index was measured by the continuous thermodilution cardiac output technique. Meanwhile, the Child–Pugh score and the model for end-stage liver disease (MELD) score were also enrolled preoperatively.

Previous studies have indicated that intraoperative events have a crucial role in the development of post-LT AKI; therefore, we intended to collect as many intraoperative variables as possible (3, 13, 14). The intraoperative data in this study included the operation time, anhepatic time, inferior vena cava occlusion time, intraoperative hypotension, estimated blood loss, fluid administration (crystalloid and colloid), intraoperative transfusion [red blood cell (RBC), fresh frozen plasma (FFP) and platelet], intraoperative drug dosage (furosemide and insulin), total urine volume and anhepatic phase urine volume.

Postoperative data included the length of postoperative hospital stay, length of ICU stay, and in-hospital mortality. In addition, postoperative laboratory data [e.g., serum creatinine and aspartate aminotransferase (AST)] were also obtained.

### Sample Size

As previously reported, the effective sample size for prediction generally suggests at least 10 events per variable, where events are defined as the proportion of cases in the least frequent of two outcome categories (15, 16). To our knowledge, the prevalence of AKI after LT, according to the KDIGO criteria, has been reported to be approximately 40%–60%, (4, 5) and we expected a 50% prevalence in our institution. Considering the accuracy and practicability of the nomogram model, 6 or fewer variables is thought to be appropriate, and therefore, 120 patients or more were required after calculation in the training set.

### Outcome and Definition

All LT patients underwent a subcostal incision and midline extension under general anesthesia. The classic orthotopic LT was performed in the majority of patients, and we did not apply the veno-venous bypass technique during the anhepatic stage. The main outcome was the occurrence of AKI. Postoperative AKI was defined according to the 2012 KDIGO criteria as previously described, with follow-up limited to the first 7 postoperative days (17). AKI stage was determined for each patient using serum creatinine concentration- and urine output-based KDIGO definitions for stage 1 (≥0.3 mg/dL or 1.5-fold increase; <0.5 ml kg^−1^ h^−1^ for 6–12 h), stage 2 (2-fold increase; <0.5 ml kg^−1^ h^−1^ for ≥12 h) and stage 3 (≥4 mg/dL or 3-fold increase). The baseline serum creatinine was tested on the day before surgery. As any degree of AKI is associated with increased risk-adjusted mortality, we decided to include all stages of AKI in this study rather than only choosing severe AKI (stages 2 and 3), as previously reported (2, 7, 8, 18, 19). Hypertension and diabetes mellitus were defined according to standard criteria (20, 21). The definition of kidney diseases before surgery was kidney function damage or an estimated glomerular filtration rate (eGFR) < 60 ml/min/1.73 m^2^. The MELD score was calculated based on the validated logarithmic index of serum bilirubin, creatinine and international normalized ratio (INR) as previously described (22). Intraoperative hypotension was defined as a systolic blood pressure <90 mmHg, a mean blood pressure <60 mmHg and/or a reduction in systolic blood pressure > 40 mmHg from baseline, as well as a duration greater than 15 min (23).

### Statistical Analysis

All statistical analyses were performed with R software (version 4.2.0) and SPSS (version 23.0.0.0). Continuous variables are expressed as medians and interquartile ranges, while categorical variables are shown as case numbers and percentages. We applied the Mann-Whitney U test to compare the median of a continuous variable between two groups. To evaluate categorical data between two independent groups, a Chi-square test was used. A least absolute shrinkage and selection operating (LASSO) regression model was performed to select the optimal predictive factors, and the lambda within 1 standard error of the minimum was applied in this study. Those non-zero coefficients factors were then incorporated into the multivariable logistic regression model (24). A predictive nomogram model was developed with the training set based on the results of the multivariable logistic regression analysis. Subsequently, the performance of the model was evaluated by calibration and discrimination in the training and validation sets (25). The calibration ability of the predictive model was used by calibration curves (26). The discriminative ability of the model was determined by the area under the receiver operating characteristic (ROC) curve (AUC) (27, 28). Decision curve analysis (DCA) was also used to evaluate the clinical application value of this predictive model (29). In addition, an interactive web-based calculator based on this nomogram model was programmed with the Shiny package (https://CRAN.R-project.org/package=shiny). For analyses, a *p*-value < 0.05 was considered to be statistically significant in a 2-tailed test.

## Results

### Overall Characteristics of Patients

**FIGURE 1:**
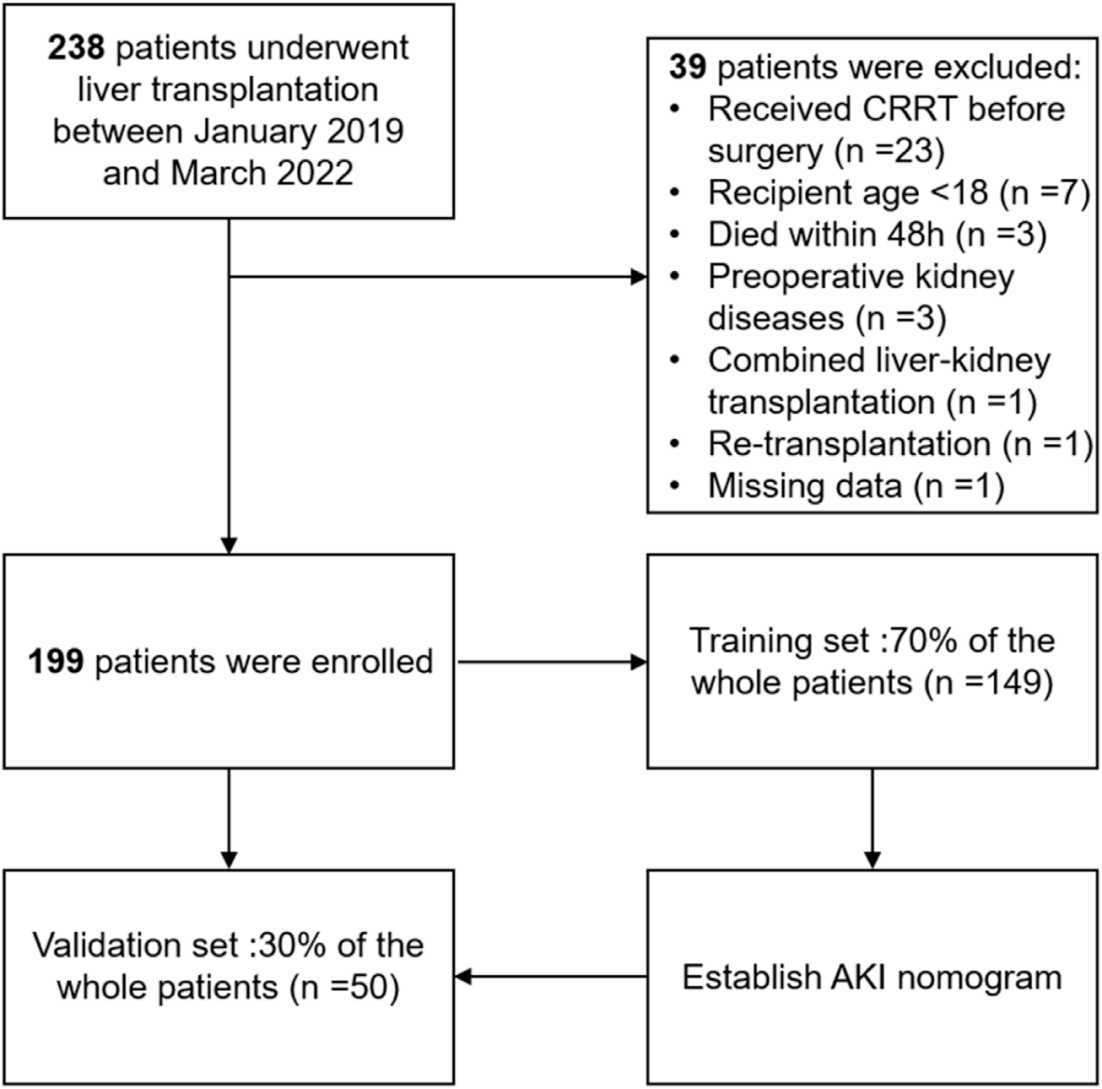
The flowchart of our study.

From January 2019 to March 2022, a total of 238 patients who underwent LT in our hospital were enrolled. According to the inclusion criteria, a total of 199 patients were identified in the final analysis, including 149 patients in the training set and 50 patients in the validation set, and the detailed flowchart is provided in Figure 1. The overall characteristics of patients in the two sets are summarized in Table 1. Fifty-three (37.9%) and twenty-one (35.6%) patients developed postoperative AKI in the training and validation cohorts, respectively.

**TABLE 1:** Clinical characteristics of the study population in the training and validation sets.

Variables	Number (percentage, %) or median (IQRs)	
	Training set (*n* = 140)	Validation set (*n* = 59)
AKI after LT, yes	53 (37.9)	21 (35.6)
Sex, male/female	126/14 (90/10)	48/11 (81.4/18.6)
Age, <65/≥65 years old	124/16 (88.6/11.4)	54/5 (91.5/8.5)
BMI, <28/≥28 kg/m^2^	126/14 (90/10)	51/8 (86.4/13.6)
ASA score, Ⅲ/Ⅳ/Ⅴ	43/91/6 (30.7/65/4.3)	16/40/3 (27.1/67.8/5.1)
Smoking	36 (25.7)	11 (18.6)
Alcoholism	38 (27.1)	14 (23.7)
Etiology of liver disease		
HBV hepatitis	57 (40.7)	24 (40.7)
HBV hepatitis + liver cancer	50 (35.7)	21 (35.6)
Liver cancer	12 (8.57)	2 (3.4)
Others	21 (15)	12 (20.3)
Preoperative comorbidities		
Diabetes mellitus	15 (10.7)	8 (13.6)
Hypertension	21 (15)	9 (15.2)
Cardiovascular diseases	7 (5)	2 (3.4)
Respiratory diseases	19 (13.6)	7 (11.9)
Previous abdominal surgery	58 (41.4)	18 (30.5)
Preoperative scores		
MELD score	13 (9–17)	13 (9–19)
Child-Pugh score	8 (6–9)	8 (6–10)
Encephalopathy	14 (10)	9 (15.2)
Ascites		
No	16 (11.4)	7 (11.9)
Non-severe ascites	109 (77.9)	45 (76.3)
Severe	15 (10.7)	7 (11.9)
Preoperative laboratory data		
WBC count, <4/4–10/>10 ×10^9^/L	66/70/4 (47.1/50/2.9)	28/30/1 (47.5/50.8/1.7)
Neutrophils count, <1.8/1.8–6.3/>6.3 ×10^9^/L	37/91/12 (26.4/65/8.6)	16/38/5 (27.1/64.4/8.5)
Lymphocyte count, <1.1/1.1–3.2/>3.2 ×10^9^/L	101/38/1 (72.2/27.1/0.7)	38/20/1 (64.4/33.9/1.7)
RBC count, <3.5/3.5–5.5/>5.5 ×10^12^/L	70/69/1 (50/49.3/0.7)	29/28/2 (49.2/47.5/3.2)
Hb level, g/L	106 (86.8–134.5)	111 (85–131.5)
Hct, <40%	103 (73.6)	45 (76.3)
PLT count, <40/40–100/>100 ×10^9^/L	26/74/40 (18.6/52.9/28.5)	11/37/11
TP, <60 g/L	37 (26.4)	12 (20.3)
ALB, <35 g/L	70 (50)	23 (38.9)
TBIL, >21 μmol/L	110 (78.6)	47 (79.7)
DBIL, >6.8 μmol/L	107 (76.4)	48 (81.4)
IBIL, >17 μmol/L	93 (66.4)	39 (66.1)
AST, >40 U/L	81 (57.9)	38 (64.4)
ALT, >50 U/L	28 (20)	12 (20.3)
BUN, >7.2 mmol/L	24 (17.1)	9 (15.3)
sCr, >133 μmol/L	3 (2.1)	0 0)
PT, >14 s	100 (71.4)	43 (72.9)
INR, >1.15	100 (71.4)	42 (71.2)
aPTT, >32 s	85 (60.7)	40 (67.8)
TT, >21 s	34 (24.3)	12 (20.3)
FIB, ≤4 g/L	134 (95.7)	58 (98.3)
Potassium, <3.5/3.5–5.5/>5.5 mmol/L	25/115/0 (17.9/82.1/0)	13/45/1 (22/76.3/1.7)
Sodium, <135/135–145/>145 mmol/L	35/104/1 (25/74.3/0.7)	13/46/0 (22/78/0)
Chlorine, <100/100–110/>110 mmol/L	30/103/7 (21.4/73.6/5)	14/45/0 (23.7/76.3/0)
Preoperative CVP, cmH_2_O	9 (7–12)	9 (8–13)
Preoperative CO, L/min	7 (5.4–8.4)	7 (5.9–8.1)
Preoperative eGFR, (mL/min/1.73 m^2^)	99.4 (78.2–124.2)	100.1 (79.7–131.1)
Intraoperative data		
Operation time, minutes	425 (383.5–475)	443 (401.5–499)
Anhepatic time, minutes	60 (51.8–70)	64 (52–74.5)
IVC occlusion time, minutes	57.5 (47–63.2)	59 (48–69.5)
Intraoperative hypotension	45 (32.1)	17 (28.8)
Estimated blood loss, mL	600 (400–1,000)	600 (400–1,000)
Fluid administration		
Crystalloid, mL	2000 (1,200–2,800)	1875 (1,050–2,700)
Colloid, mL	300 (200–862.5)	300 (200–725)
Intraoperative transfusion		
RBC, U	4 (0–7.63)	4 (1.5–6.8)
FFP, ml	600 (200–1,000)	600 (400–900)
PLT, U	1 (0–1)	1 (0–1)
Intraoperative drugs dosage		
Furosemide, mg	20 (0–20)	20 (0–35)
Insulin, IU	35 (0–60)	20 (0–80)
Total urine volume, ml	1,000 (697.5–1,512.5)	1,000 (725–1,375)
Anhepatic phase urine volume, ml	65 (20–200)	69 (40–150)

Abbreviations: IQR, interquartile range; AKI, acute kidney injury; LT, liver transplantation; BMI, body mass index; ASA, American Society of Anesthesiologists; HBV, hepatitis B virus; MELD, model for end-stage liver disease; WBC, white blood cell; RBC, red blood cell; Hb, hemoglobin; Hct, hematocrit; PLT, platelet; TP, total protein; ALB, albumin; TBIL, total bilirubin; DBIL, direct bilirubin; IBIL, indirect bilirubin; ALT, alanine aminotransferase; AST, aspartate aminotransferase; BUN, blood urea nitrogen; sCr, creatinine; PT, prothrombin time; INR, international normalized ratio; aPTT, activated partial thromboplastin time; TT, thrombin time; FIB, fibrinogen; CVP, central venous pressure; CO, cardiac output; eGFR, estimated glomerular filtration rate; IVC, inferior vena cava; FFP, fresh frozen plasma.

To analyze the postoperative characteristics and outcomes, we divided the patients into two groups (AKI and non-AKI groups) (Table 2). The AKI group had a longer length of ICU stay (*p* < 0.001) and higher peak AST (*p* < 0.001) and peak serum creatinine levels (*p* = 0.049) than the non-AKI group. Notably, the in-hospital mortality of the AKI group was approximately 10 times greater than that of the non-AKI group (*p* = 0.003).

**TABLE 2:** Postoperative characteristics and outcomes of the patients in AKI and non-AKI groups.

Variables	Number (percentage, %) or median (IQRs)		*p*-value
	AKI (*n* = 74)	Non-AKI (*n* = 125)	
Peak AST (U/L)	1326.5 (613.3–3459.5)	1,026 (591–1752)	<0.001
Peak sCr, μmol/L	136 (105–188.3)	93 (77–110)	0.049
Postoperative hospital stay (day)	26 (21–40.3)	25 (18–31)	0.057
Length of ICU stay (day)	7 (5–10)	5 (4–6)	<0.001
Died in hospital	7 (9.46)	1 (0.8)	0.003

Continuous variables are expressed as medians and interquartile ranges, while categorical variables are showed as case numbers and percentages. Abbreviations: AST, aspartate aminotransferase; sCr, creatinine; ICU, intensive care unit.

### Variable Selection of AKI After LT

**FIGURE 2:**
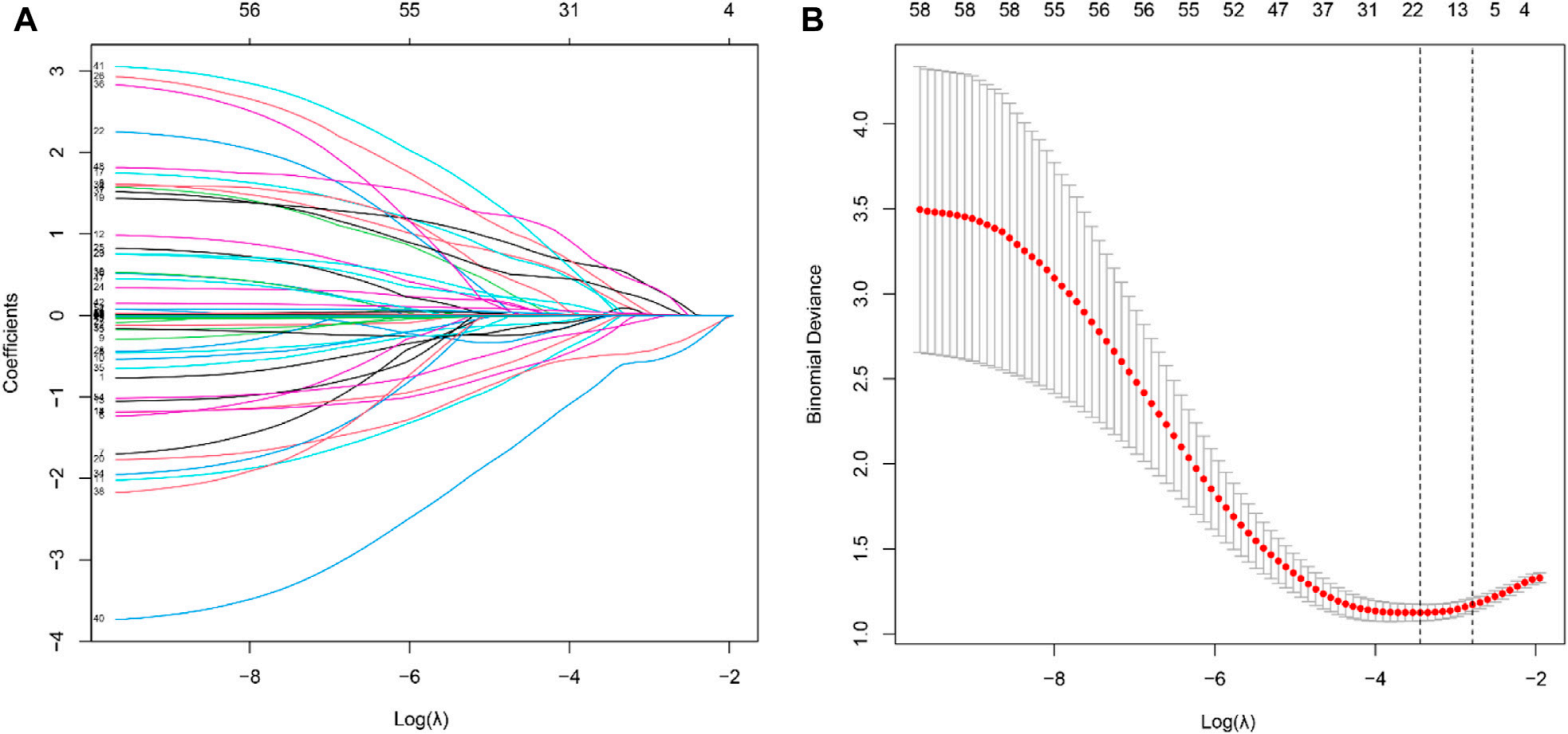
Feature selection of LT patients using the LASSO logistic regression model. **(A)** A Lasso coefficient profile plot was built for the prediction of AKI after LT. **(B)** The optimal parameter (*λ*) was selected by the LASSO model using 10-fold cross-validation *via* 1 standard error of the minimum criteria.

Using the LASSO algorithm, fifty-eight variables were reduced to 9 potential features, with a cross-validated error within 1 standard error of the minimum (Figures 2A, B). Five factors remained independently associated with the risks of AKI in LT patients after multivariate logistic regression analysis (Table 3). Preoperative variables (whole blood serum lymphocyte count, RBC count and serum sodium) and intraoperative variables (insulin dosage and anhepatic phase urine volume) were independent risk factors for post-LT AKI.

**TABLE 3:** Multivariable logistic regression analysis of predicting AKI after LT in the training cohort.

Variables	*β*	OR (95% CI)	*p*-value
Intercept	−0.397	0.673 (0.152–2.928)	0.596
Lymphocyte count, ×10^9^/L	1.150	3.159 (1.496–7.031)	0.003
RBC count, ×10^12^/L	−0.875	0.417 (0.185–0.904)	0.029
TT, s	0.596	1.815 (0.794–4.181)	0.157
Sodium, mmol/L	−1.108	0.330 (0.138–0.762)	0.010
Preoperative CVP, cmH_2_O	0.055	1.056 (0.968–1.162)	0.234
Intraoperative hypotension	1.505	4.506 (0.755–33.105)	0.110
Intraoperative furosemide dosage, mg	0.018	1.018 (1.000–1.038)	0.059
Intraoperative insulin dosage, IU	0.009	1.009 (1.002–1.019)	0.016
Anhepatic phase urine volume, ml	−0.009	0.991 (0.986–0.996)	<0.001

*β* is the regression coefficient. Abbreviations: AKI, acute kidney injury; LT, liver transplantation; OR, odds ration; CI, confidence interval; RBC, red blood cell; TT, thrombin time; CVP, central venous pressure.

### Model Construction and Online Calculator Development

**FIGURE 3:**
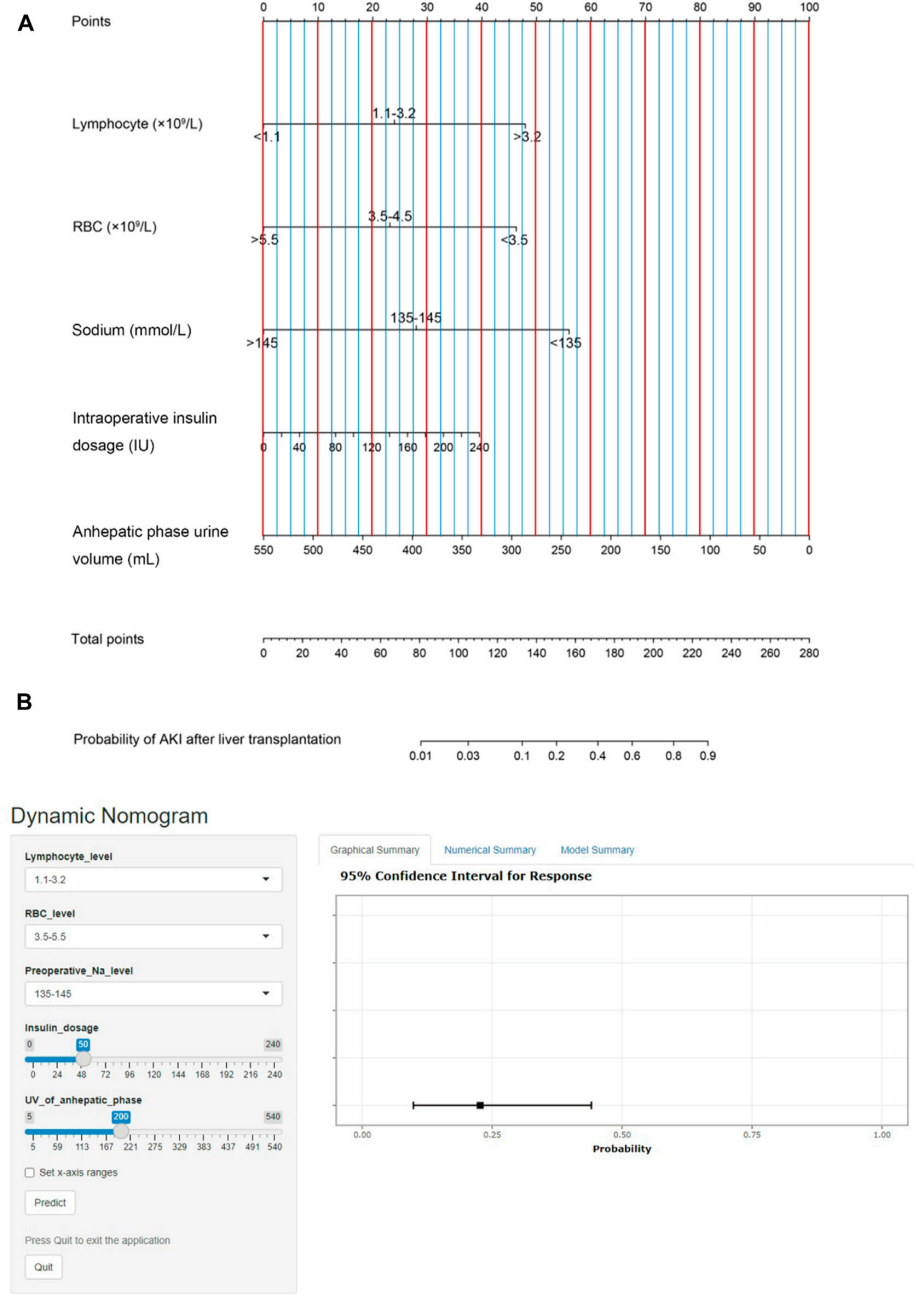
**(A)** The post-LT AKI nomogram model was developed. This nomogram was constructed in the training set, with whole blood serum lymphocyte count, RBC count, sodium, intraoperative insulin dosage and anhepatic phase urine volume. **(B)** Online calculator predicting acute kidney injury after liver transplantation accessible at https://caobingbing.shinyapps.io/Nomogram_for_AKI_after_liver_transplantation/, depicting an example for predicting the probability of an LT patient with normal preoperative whole blood serum lymphocyte count, RBC count and serum sodium. The intraoperative insulin dosage was 50 IU, and anhepatic phase urine volume was 200 ml. Abbreviations: AKI, acute kidney injury; LT, liver transplantation; RBC, red blood cell.

We then established a simple-to-use nomogram model based on the above independent risk factors (Figure 3A). The nomogram showed that anhepatic phase urine volume was the most significant predictor for post-LT AKI, followed by preoperative serum sodium and whole blood lymphocyte count. To make this model more convenient for clinicians, we also developed an online calculator (Figure 3B). This online calculator is available at https://caobingbing.shinyapps.io/Nomogram_for_AKI_after_liver_transplantation/.

### Performance and Validation of the Post-LT AKI Nomogram Model

**FIGURE 4:**
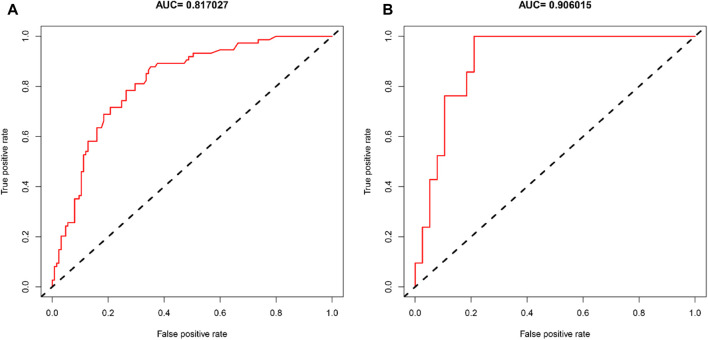
Diagnostic accuracy of the post-LT AKI nomogram model in the training and validation sets. ROC curves showed AUCs of 0.817 and 0.906 in the training **(A)** and validation sets **(B)**.

**FIGURE 5:**
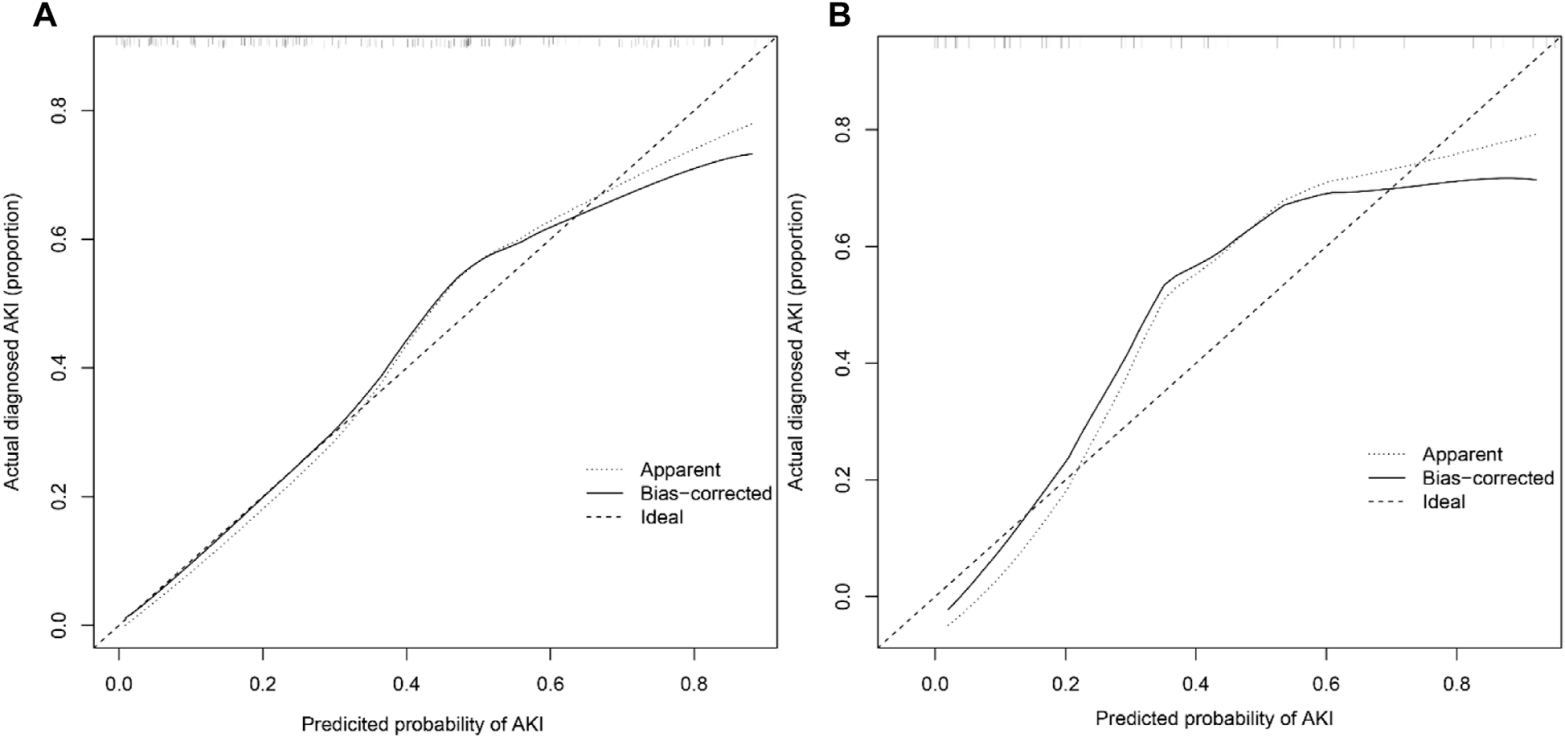
Calibration curves of the post-LT AKI nomogram model in the training **(A)** and validation sets **(B)** (Bootstrap = 1,000 repetitions).

The AUCs of the predictive nomogram were 0.817 (95% CI: 0.758–0.876) and 0.906 (95% CI: 0.831–0.981) for the training and validation sets, respectively (Figures 4A, B), indicating that this model demonstrates excellent accuracy in estimating the probability of AKI after LT. The calibration curve of the nomogram is presented in Figures 5A, B, which revealed good consistency between prediction and observation.

### Clinical Use of the Post-LT AKI Nomogram Model

**FIGURE 6:**
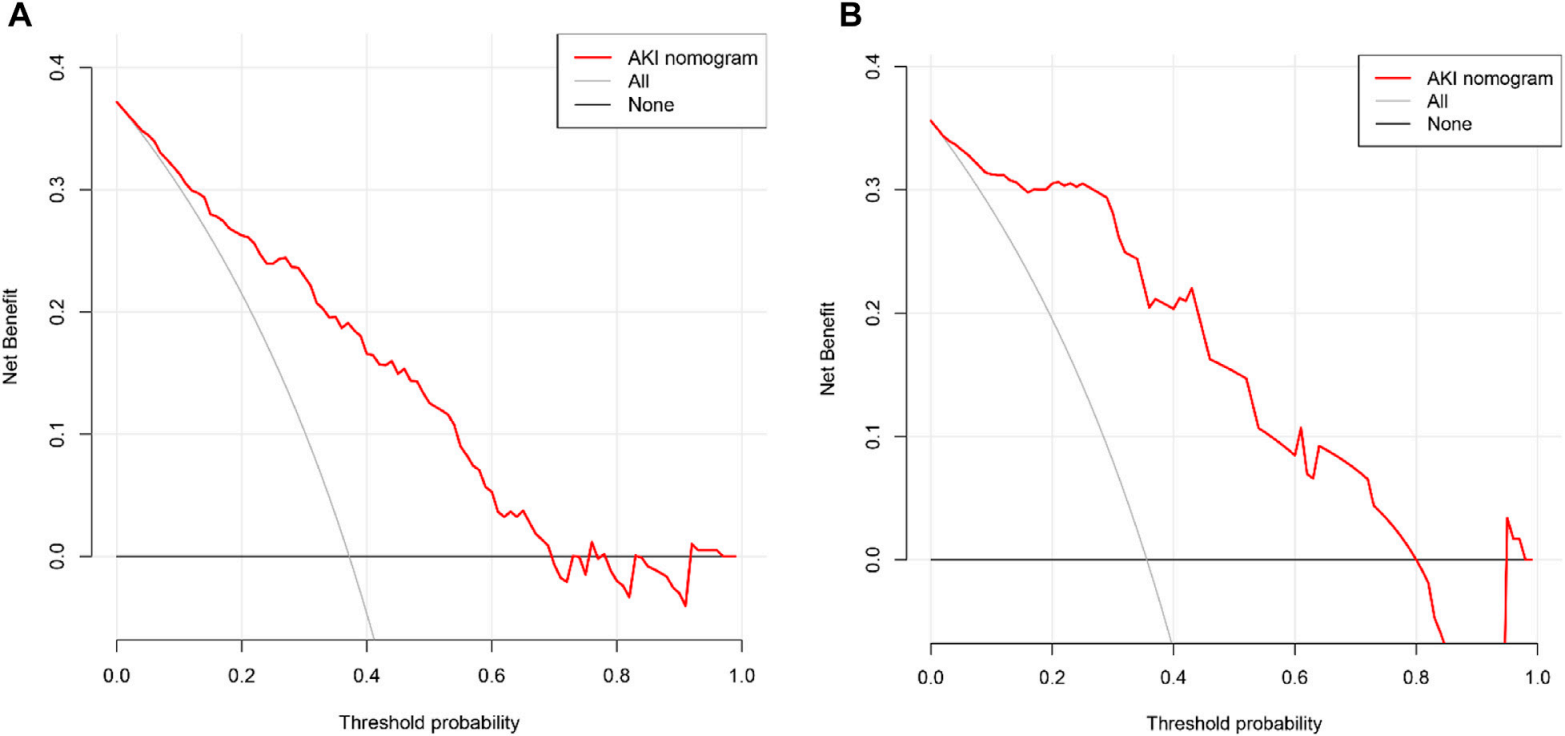
Decision curve analysis of the nomogram for the estimation of post-LT AKI in the training set **(A)** and validation set **(B)**.

Finally, the DCA of AKI after the LT nomogram model is presented in Figures 6A, B. Compared with scenarios where no prediction model was used for a pretreatment decision, the nomogram model provided a favorable net benefit across a wide range, with a threshold probability across 1%–70% and 1%–80% for the training and validation sets, respectively. Overall, the above methods confirmed the clinical utility and reliability of our nomogram.

## Discussion

Herein, we describe a nomogram model to predict the probability of AKI in patients who underwent LT and then developed an online calculator for clinical use. In this study, we identified five risk factors as independent predictors for post-LT AKI, including whole blood serum lymphocyte count, RBC count, serum sodium, intraoperative insulin dosage and anhepatic phase urine volume. Moreover, we obtained several new findings in this study. First, the post-LT AKI nomogram model is a useful tool with good discrimination and calibration. Second, the AKI risk of individual patients who underwent LT can be calculated easily at the end of transplantation surgery by using our online calculator. Once clinicians identify high-risk patients, renal protection treatment (e.g., avoiding nephrotoxic medications and adjusting the dose of calcineurin inhibitors) is applied in a timely manner. Third, at a threshold probability between 1% and 70%, using our nomogram model may add more benefit to LT patients, indicating that the model described here may be widely applicable.

In the current study, we noted that AKI is a common issue for LT patients. According to the 2012 KDIGO criteria, we observed that the incidence of post-LT AKI in our center was 37.2%, which is slightly lower than the 40%–60% reported previously (4, 5, 7, 8). However, compared with the other surgical population (e.g., orthopedic and gastrointestinal surgery), which was reported to vary from 2.9% to 11.8%, LT patients have a much higher postoperative AKI incidence. (30, 31) In addition, we also observed that the in-hospital mortality of the AKI group was approximately 10 times greater than that of the non-AKI group. The length of ICU stay was significantly increased in the AKI group. Therefore, as reported in previous studies, we similarly demonstrate that post-LT AKI may lead to serious complications and cause worse prognosis (2, 4, 10, 13, 19, 30).

Many studies have demonstrated that several intraoperative factors can significantly contribute to postoperative AKI (14), and electrolyte/acid-base balance disorder, hypotension, blood or FFP transfusion, lactate concentration and urine output have been associated with an increased incidence of AKI. (4, 5, 7, 8) As we expected, two intraoperative risk factors (insulin dosage and anhepatic phase urine volume) were identified as independent predictors in this study. Regarding such intraoperative factors, clinicians can modify our intraoperative care and improve surgical procedures to reduce the risk of postoperative AKI.

The strongest risk factor in the nomogram model was anhepatic phase urine volume, which has not been reported before. The anhepatic phase is a unique and pivotal time during liver transplantation, and acid-base balance disorder, hemodynamic instability and renal congestion are much more pronounced (1, 14). Xu and colleagues (32) reported that low urine volume was significantly related to AKI, which reflects inadequate renal perfusion. A recent study demonstrated that an intraoperative urine volume < 0.5 ml kg^−1^ h^−1^ was associated with postoperative AKI in a major abdominal surgery population (33). Therefore, based on the results of our study, we can assume that a patient with more urine volume of the anhepatic phase during surgery may have better early renal function and a lower risk of postoperative AKI. Although there is debate about intraoperative urine volume reflecting renal function, we did provide a new idea for post-LT AKI. Further study is essential 1) to determine the potential association between anhepatic phase urine volume and AKI in LT patients; 2) to identify an optimal threshold of anhe-patic phase urine volume for predicting the postoperative AKI risk in patients receiving LT.

Another intraoperative factor we found in this study is the dosage of insulin, which is routinely used to maintain normoglycemia and correct acidosis. The intraoperative insulin requirement might reflect hyperglycemia and severe acid-base balance disorder, which can cause hypercoagulability, oxidative stress and endothelial dysfunction. As reported before, perioperative hyperglycemia has been suggested as a risk factor for post-LT AKI and is known to be associated with adverse outcomes in all inpatients, especially in sepsis (2). In this study, we found that insulin dosage, which has a positive correlation with AKI risk, is an important risk factor in LT patients and contributes to the development of post-LT AKI.

Three preoperative risk factors were identified as independent predictors, including whole blood lymphocyte count, RBC count and serum sodium. In general, the whole blood lymphocyte count is considered an indicator of the immune response in patients (34). In addition, lymphocytes, especially T cells, play a vital role in the whole evolution of kidney injury (35). In this study, we found that a higher whole blood lymphocyte count can increase the risk of post-LT AKI, rather than a low whole blood lymphocyte count. The possible explanation for this result may be that both dysregulation or overactivation of the immune response contribute to renal damage. Besides, the hepatitis infection preoperatively and the use of immunosuppressive medications during surgery have potential effect on the level of lymphocyte count. The preoperative whole blood RBC count was also significantly associated with post-LT AKI. The development of RBCs is regulated by erythropoietin, which is mainly produced by the kidneys, and the level of erythropoietin is de-creased in renal dysfunction patients. As preoperative renal dysfunction was previously reported as an independent predictor for post-LT AKI, we hypothesized that the patients in our study may have potential renal dysfunction (36). Meanwhile, a lower RBC count may be related to a higher risk of intraoperative hemorrhage and blood transfusion, which have been identified as risk factors for post-LT AKI. Overall, the fact that reduced RBC count is an interesting observation needing further studies to help us better understand the etio-pathogenesis of AKI. Additionally, we found that serum sodium was independently associated with post-LT AKI, which is consistent with a previous study (37). For patients awaiting liver transplantation, hyponatremia is associated with an increased frequency of complications (e.g., AKI) and reduced short-term survival (38). The occurrence of preoperative hyponatremia is often related to renal impairment, which is characterized by a decreased capacity for solute-free water excretion. Although the MELD score and recipient BMI have been previously reported to predict post-LT AKI, our study failed to show any association with postoperative AKI. This may be the result of our population having relatively low MELD scores, and the majority of recipients were not obese.

Measures should be taken to prevent AKI after identifying high-risk patients. One proposal to decrease the incidence of post-LT AKI is perioperative renal protection, such as promoting hemodynamic stability during the anhepatic phase (2). Another important measure is to reduce hepatic IRI, although this can be a difficult task. Extracorporeal donor liver perfusion is a new approach to reduce hepatic IRI and has been shown to be feasible (39). Taken together, this is not the first prediction nomogram model for post-LT AKI; however, it is the first to develop an online calculator for clinical use, and the discrimination and calibration of the model are excellent. Importantly, we applied the 2012 KDIGO criteria to define AKI instead of the old Risk, Injury, Failure, Loss, and End-stage criteria.

Our current study had several limitations. First, information on long-term survival was not available in this study, and this part of the work is now in progress. Additionally, due to the privacy protection policy, we failed to include donor data from electronic medical records. Next, selection bias may exist, as our study had a retrospective design. Finally, external validation in other populations or countries is needed to demonstrate its applicability. Indeed, a larger, more heterogeneous cohort at our institution is planned in the future to identify more predictors.

In conclusion, we present and validate an easy-to-use nomogram model to predict the probability of AKI in LT patients and then describe an online calculator to predict the AKI risk of each individual patient. This predictive model, including preoperative and intraoperative predictors, is a useful tool with good discrimination and calibration. The great advantage of our model is that it is available for clinicians to identify high-risk AKI patients at the end of surgery and avoid unnecessary postoperative renal injury.

## Data Availability

The raw data supporting the conclusion of this article will be made available by the authors, without undue reservation.

## Glossary

AKI: acute kidney injury
ALT: alanine aminotransferase
ALB: albumin
aPTT: activated partial thromboplastin time
ASA: American Society of Anesthesiologists
AST: aspartate aminotransferase
AUC: area under the receiver operator characteristic curve
BMI: body mass index
BUN: blood urea nitrogen
CO: cardiac output
CRRT: continuous renal replacement therapy
CVP: central venous pressure
DBD: deceased after brain death
DCA: decision curve analysis
eGFR: estimated glomerular fil-tration rate
FFP: fresh frozen plasma
GB: globulin
Hb: hemoglobin
HBV: hepatitis B virus
Hct: hematocrit
ICU: intensive care unit
INR: international normalized ratio
IQR: interquartile range
IRI: ischemia reperfusion injury
IVC: inferior vena cava
KDIGO: Kidney Disease Improving Global Outcomes
LASSO: least absolute shrinkage and selection operator
LT: liver transplantation
MELD: model for End-Stage Liver Disease
PLT: platelet
PT: prothrombin time
RBC: red blood cell
ROC: receiver operator characteristic
sCr: creatinine
TBil: total bilirubin
TP: total protein
TT: thrombin time
WBC: white blood cell
